# Whole-brain connectivity analysis and classification of spinocerebellar ataxia type 7 by functional MRI

**DOI:** 10.1186/2053-8871-1-2

**Published:** 2014-06-16

**Authors:** Carlos R Hernandez-Castillo, Víctor Galvez, Consuelo Morgado-Valle, Juan Fernandez-Ruiz

**Affiliations:** Departamento de Fisiología, Facultad de Medicina, Universidad Nacional Autonoma de Mexico, Distrito Federal C P., 04510 Mexico; Posgrado en Neuroetologia, Universidad Veracruzana, Xalapa, Mexico; Instituto de Investigaciones Cerebrales, Universidad Veracruzana, Xalapa, Mexico; Facultad de Psicologia, Universidad Veracruzana, Xalapa, Mexico

**Keywords:** SCA, MVPA, Resting state, Functional connectivity, Spinocerebellar ataxia

## Abstract

**Background:**

Spinocerebellar ataxia type 7 (SCA7) is a genetic disorder characterized by degeneration of the motor and visual systems. Besides neural deterioration, these patients also show functional connectivity changes linked to the degenerated brain areas. However, it is not known if there are functional connectivity changes in regions not necessarily linked to the areas undergoing structural deterioration. Therefore, in this study we have explored the whole-brain functional connectivity of SCA7 patients in order to find the overall abnormal functional pattern of this disease. Twenty-six patients and age-and-gender-matched healthy controls were recruited. Whole-brain functional connectivity analysis was performed in both groups. A classification algorithm was used to find the discriminative power of the abnormal connections by classifying patients and healthy subjects.

**Results:**

Nineteen abnormal functional connections involving cerebellar and cerebral regions were selected for the classification stage. Support vector machine classification reached 92.3% accuracy with 95% sensitivity and 89.6% specificity using a 10-fold cross-validation. Most of the selected regions were well known degenerated brain regions including cerebellar and visual cortices, but at the same time, our whole-brain connectivity analysis revealed new regions not previously reported involving temporal and prefrontal cortices.

**Conclusion:**

Our whole-brain connectivity approach provided information that seed-based analysis missed due to its region-specific searching method. The high classification accuracy suggests that using resting state functional connectivity may be a useful biomarker in SCA 7.

**Electronic supplementary material:**

The online version of this article (doi:10.1186/2053-8871-1-2) contains supplementary material, which is available to authorized users.

## Background

Spinocerebellar ataxia 7 (SCA7) is an Autosomal Dominant Cerebellar Ataxia (ADCAs) caused by the expansion of the cytosine-adenine-guanine (CAG) trinucleotide in the codon region of the chromosome 3p21 encoding the protein ataxin 7 [1]. SCA7 is considered one of the rarest forms of genetic ADCAs [2]. Clinically, SCA7 is characterized by the combination of cerebellar ataxia and macular degeneration and is the only spinocerebellar ataxia that causes permanent blindness [3–5]. The brain degeneration associated with SCA7 has been relatively well documented, featuring severe neuronal loss in a broad range of cerebellar and cerebral regions [6–11]. Previous work using resting state fMRI (rsfMRI) to explore the effect of such degeneration on the pattern of functional connectivity found hyper/hypo connectivity changes between degenerate and non-degenerate areas [12].

By measuring the temporal synchronization among distant brain areas, the rsfMRI technique [13] proves to be a powerful tool for delineating the brain’s functional connectivity and has been successfully applied to study functional disruption patterns of intrinsic neural networks in various neurodegenerative disorders [14, 15]. These abnormal patterns can be helpful for improving our understanding of the pathophysiological mechanisms underlying neurodegenerative disorders and might represent a possible functional biomarker to measure the effects of putative therapeutic approaches. Using different methods, several studies have used this new information to classify brain disorders such as Alzheimer’s disease, mild cognitive impairment, major depression and autism, among others [16–20]. In recent years, there has been an increasing interest in using multivariate pattern analysis methods to distinguish patients from healthy controls by means of structural or functional brain images [16, 21–26], where the use of support vector machines (SVM) [27] arises as the most popular classifier due to its good performance and reliability against noise [18, 28].

In this study, we systematically delineated the functional changes associated with SCA7 using a whole-brain approach in 26 SCA7 patients. Then, we used the abnormal pattern of functional connectivity as a classification feature to discriminate between patients and healthy controls. Based on our previous work [12], we expected that the most discriminative functional connections would be between the cerebellum and the visual and motor cortices, however, a whole-brain approach could reveal new information about other brain regions not explored before.

## Results

### Abnormal functional connections

**Figure 1:**
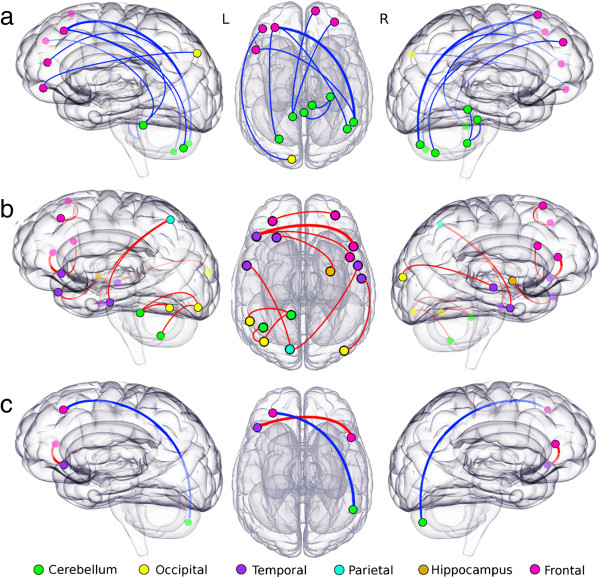
**Highest differences between patients with SCA7 and healthy controls.** Nodes represent the AAL regions involved in each of 19 abnormal functional connections. Node color indicates the anatomical location and line color indicates the abnormality. Row **a)** shows the connection with decreased functional connectivity, row **b)** shows the connections with increased functional connectivity and row **c)** indicates two connections showing the higher differences between SCA7 and healthy control (p < 0.0001, see Table 1). Note that for side views translucent nodes are located in the opposite hemisphere.

Nineteen abnormal functional connections met our threshold criteria. These included regions in the bilateral cerebellum, inferior/middle/superior temporal gyri, left precuneus, left occipital gyrus, left fusiform gyrus and inferior/middle/superior frontal gyrus (Table 1 and Table 2). The most affected functional connections in SCA7 were a hypoconnectivity between the right cerebellum crus II and the left middle frontal gyrus and a hyperconnectivity between the left superior temporal pole and the right inferior frontal gyrus in the triangular part. See Figure 1 for a representative image of selected connections. Moreover, our analysis revealed a synchrony decrease within the cerebellar cortex and between cerebellar and frontal regions, as well as a synchrony increase between temporal and several brain regions including precuneus, hippocampus and middle occipital and inferior frontal gyri (Table 3). The connection between the left middle frontal gyrus and the right superior frontal gyrus showed a negative correlation with the Scale for the Assessment and Rating of Ataxia (SARA) score and the symptoms onset (Additional file 1: Figure S1).

**Table 1:** **Demographic information of SCA7 group**

ID	Age	Gender	Years of symptoms	CAG expansion	SARA
P01	40	F	21	50	27
P02	44	F	6	44	9
P03	68	F	1	50	6
P04	43	F	21	47	15
P05	42	F	17	47	29.5
P06	18	M	15	50	19.5
P07	39	F	23	50	27
P08	18	F	4	53	7
P09	19	M	7	71	29.5
P10	34	M	10	55	17
P11	35	M	14	52	16
P12	64	M	10	43	14.5
P13	47	M	6	50	13
P14	23	M	3	61	12.5
P15	52	M	7	46	12
P16	44	M	4	48	11
P17	40	F	13	55	23
P18	60	M	6	45	16
P19	54	M	6	43	24
P20	45	F	7	44	12
P21	35	F	1	42	8.5
P22	21	F	1	46	4
P23	20	M	1	48	4
P24	30	M	6	48	12
P25	61	M	7	41	10.5
P26	29	M	7	48	26

**Table 2:** **Functional connections showing abnormal connectivity pattern in SCA7**

Automated anatomical labeling brain regions		P-value	Abnormality
Right cerebellum crus II	Left Middle Frontal Gyrus	0.00005	Decrease
Left superior temporal pole	Right Inferior Triangular Frontal Gyrus	0.00005	Increase
Right cerebellum 10	Right Cerebellum 3	0.00011	Decrease
Left cerebellum 9	Left Fusiform Gyrus	0.00016	Increase
Left inferior temporal gyrus	Left Precuneus	0.00023	Increase
Right cerebellum crus II	Left Inferior Triangular Frontal Gyrus	0.00027	Decrease
Left middle temporal pole	Right Hippocampus	0.00034	Increase
Right middle temporal gyrus	Right Middle Occipital Gyrus	0.00034	Increase
Left cerebellum crus II	Left Middle Frontal Gyrus	0.00048	Decrease
Left cerebellum 9	Left Inferior Occipital Gyrus	0.00048	Increase
Left middle frontal gyrus	Right Superior Frontal Gyrus	0.00050	Increase
Left cerebellum 9	Right Superior Frontal Gyrus	0.00053	Decrease
Vermis 3	Right Cerebellum 10	0.00055	Decrease
Right inferior temporal gyrus	Left Precuneus	0.00060	Increase
Left superior temporal pole	Right inferior Opercular Frontal Gyrus	0.00062	Increase
Right cerebellum 7	Left Middle Frontal Gyrus	0.00065	Decrease
Left cerebellum 7	Left Inferior Occipital Gyrus	0.00069	Increase
Left cuneus	Left Inferior Frontal Gyrus	0.00069	Decrease
Left cerebellum 9	Right Medial Superior Frontal Gyrus	0.00094	Decrease

**Table 3:** **SCA7 abnormal functional connections sorted by anatomical regions**

Automated anatomical labeling anatomical regions		Abnormality
**Vermis 3**	**Right Cerebellum 10**	**Decrease**
**Right cerebellum 10**	**Right Cerebellum 3**	**Decrease**
Left cerebellum 9	Left Fusiform Gyrus	Increase
Left cerebellum 9	Left Inferior Occipital Gyrus	Increase
Left cerebellum 7	Left Inferior Occipital Gyrus	Increase
**Right cerebellum 7**	**Left Middle Frontal Gyrus**	**Decrease**
**Right cerebellum crus II**	**Left Inferior Triangular Frontal Gyrus**	**Decrease**
**Right cerebellum crus II**	**Left Middle Frontal Gyrus**	**Decrease**
**Left cerebellum crus II**	**Left Middle Frontal Gyrus**	**Decrease**
**Left cerebellum 9**	**Right Medial Superior Frontal Gyrus**	**Decrease**
**Left cerebellum 9**	**Right Superior Frontal Gyrus**	**Decrease**
Right inferior temporal gyrus	Left Precuneus	Increase
Left inferior temporal gyrus	Left Precuneus	Increase
Left middle temporal pole	Right Hippocampus	Increase
Right middle temporal gyrus	Right Middle Occipital Gyrus	Increase
Left superior temporal pole	Right Inferior Triangular Frontal Gyrus	Increase
Left superior temporal pole	Right inferior Opercular Frontal Gyrus	Increase
**Left inferior frontal gyrus**	**Left Cuneus**	**Decrease**
Left middle frontal gyrus	Right Superior Frontal Gyrus	Increase

Bold rows indicate decreased connectivity.

### Classification results

Performance metrics reported high classification accuracy. After training the SVM classifier using the 19 abnormal functional connections previously selected, the classification accuracy reached 92.3% with 95% sensitivity and 89.6% specificity in a 10-fold cross validation.

## Discussion

In this work we explored the whole-brain functional connectivity in a large SCA7 population and demonstrated that patients can be distinguished from healthy controls using resting state fMRI with an excellent classification accuracy and sensitivity (92.3%, 95%). Moreover, our results showed that the majority of the abnormal functional connections used for classification involved regions commonly affected by SCA7 [12], including, the cerebellar and visual cortex. However, our analysis also found changes in regions not previously reported as the bilateral inferior/middle temporal gyri, right hippocampus and the triangular/opercular parts of the inferior frontal gyrus. This new information is relevant to better understand the degenerative process of SCA7. Furthermore, changes in functional connectivity might be used as potential biomarkers to test drugs that could prevent or decrease this process in the early-stage patients.

### Disruption of fronto-cerebellar network

In our previous work we found a correlation between the CAG expansion and the functional connectivity between the anterior cerebellum and the left superior frontal gyrus [12]. In that work we used a seed-based approach focused in the most degenerated regions. However, this approach restricted the search to a few specific areas. The whole-brain approach used here expands the search thorough the brain revealing a set of connections showing decreased synchrony between the cerebellum and the frontal cortex. It is well known that frontal regions are involved in motor control and planning [29–31] which also play an important role in the integration of sensory and mnemonic information, as well as regulation of intellectual function and action [32, 33]. Particularly, reduction in the metabolism of inferior/middle frontal gyri has been associated with loss of speech production, resulting in dyspraxia and dysarthria [34]. Additionally, these regions were also reported to be related to readiness and showed activity increase before the execution of self-initiated motor acts [35]. The widespread synchronicity decrease observed between the cerebellum and the frontal cortex suggests a communication disruption within the fronto-cerebellar network. Future studies should explore if the disruption of this connectivity contributes to the motor and cognitive impairments observed in these patients.

### Increase in synchrony in temporal lobes

Hyperconnectivity was found in temporal regions including inferior/middle/superior gyri. Several studies have indicated the involvement of inferior temporal cortices and precuneus in object and spatial vision [36, 37] whose abnormal functioning could produce specific disruptions in visually guided movements [38]. Moreover, the hippocampus and prefrontal regions also have been related with visual working memory [39]. Besides, these areas are also involved in language processes such as comprehension of complex semantics and encoding of concrete words [40–42]. Given previous reports that an increase in functional connectivity may allow structurally damaged brain regions to remain functional [43–45], the enhanced connectivity involving the multi-sensory integration regions observed in this study may reflect a compensating effort for the visual loss and/or speech deficits associated with SCA7 [46].

### Classification of functional connectivity

Multivariate classification of functional connectivity is gaining popularity due to good outcomes in discriminating between patients and healthy volunteers [21–26]. In this exploratory analysis we parcellated the brain by using the Automated Anatomical Labeling atlas (AAL) [47], dividing it into 116 regions based in the brain cytoarchitecture. This allowed us to retrieve the regional functional connectivity across the whole-brain. A possible issue resulting from this parcellation could be the size differences across regions. However, given the exploratory nature of this analysis, the good classification accuracy that we obtained, and the great acceptance of this atlas we believe that its use was appropriate. A different parcellation using isometric regions would mix signals from different anatomical regions, and would also increase the number of regions and therefore the noise/signal ratio. In the same way, several techniques have been proposed for the feature selection stage [48]. Our choice was simple but reported good classification levels (92.3% accuracy and 95% sensitivity), demonstrating that the connections selected were highly discriminating of this disease. Future work to classify between different types of spinocerebellar ataxia would require a finer parcellation and comprehensive feature selection.

### Limitations

In this work we used a univariate approach as a feature selection method to select a small number of high discriminative abnormal connections across the patient’s brain. The selected subset of connections reached high classification accuracy between patients and healthy controls, proving their high discriminative power. However, this approach is limited by its own nature, comparing voxel by voxel. Different alternatives try to address this problem using multivariate approaches as principal component analysis and Independent component Analysis among others. These multivariate methods convert multidimensional vectors into statistically independent components, assuming that the number of component representing the data are less than the original dimensionality reducing the data space by discarding the components with high variance [48, 49]. However, and due to this study design and the well-defined structural degeneration in SCA7 we choose to use the univariate approach. Another limitation is the lack of significant correlation with behavioral scores. Only one connection showed a significant correlation between the functional connectivity and SARA and symptoms onset. There were no significant correlations between these variables and other connections, but there were trends. This outcome can be associated to several variables, for an instance, changes in connectivity appears early in the disease progression and are followed by structural degeneration in a slow fashion [50, 51], these difference in the velocity of the progressive degeneration could affect the correlation between variables. Taking into account that SARA score measures the motor impairments as a result of cerebellar dysfunction, and these changes develop slowly compared with the connectivity changes, the absence of a good correlation between those variables is not surprising. Something similar could have happened with the CAG expansion and the symptoms onset. In future work we will address those issues by analyzing longitudinal data of the same group of patients and by using multivariate approaches as well as behavioral/clinical data, in order to better describe changes of functional connectivity over time.

## Conclusion

The use of functional connectivity measurements is a powerful tool that helps in the discrimination of neurodegenerative diseases. In this work, we demonstrated that by using whole-brain functional connectivity to classify SCA7 patients and healthy controls, a 92.3% precision accuracy was reached. At the same time our results indicate that SCA7 patients are losing synchrony within the cerebellum and between the cerebellum and different cerebral regions, like the frontal cortices. Besides, the increasing synchrony of the multi-sensory integration regions might reflect a compensatory mechanism against neurodegeneration in motor and visual systems. This outcome provides novel and relevant information about the functional changes underlying the degenerative process of SCA7 and can be helpful to better understand this rare disease. More research comparing the functional connectivity between different types of spinocerebellar ataxias will help to understand the neurodegenerative idiosyncrasies of each particular type.

## Methods

### Subjects

Twenty six patients with a molecular diagnosis of spinocerebellar ataxia type 7 [52] participated in this study (11 female, mean age 39.4, complete information for each patient is provided in Table 1). The control group consisted of 26 age- and gender-matched normal controls in absence of any neurological diseases or psychiatric disorders. Motor impairment of patients was tested using the Scale for the Assessment and Rating of Ataxia (SARA) [53]. All procedures were conducted in accordance with the international standards laid down in the 1964 Declaration of Helsinki carried out by the Institutional Committees on human experimentation. All participants gave written, informed consent before entering the study.

### Image acquisition

Images were acquired at the Instituto Nacional de Psiquiatria “Ramon de la Fuente Muñiz” using a 3.0 T Achieva MRI scanner (Phillips Medical Systems, Eindhoven, Holland). The anatomical acquisition consisted of a 3D T1 Fast Field-Echo sequence, with TR/TE = 8/3.7 ms, FOV 256 × 256 mm and an acquisition and reconstruction matrix of 256 × 256, resulting in an isometric resolution of 1 × 1 × 1 mm. Resting state fMRI images were collected using Echo Planar Imaging (EPI) single shot sequence with TR = 2000 ms, TE = 35 ms, and 120 whole-brain volumes with 34 slices. Final isometric resolution of rsfMRI images was 3 × 3 × 4 mm without gaps. During functional MRI acquisition, subjects in all groups were instructed to keep their eyes closed, to think about nothing in particular, and to stay awake. Five dummy scans were performed at the beginning of each functional acquisition to allow magnetization to reach a steady state.

### Resting state fMRI preprocessing

RsfMRI preprocessing included brain extraction, time shifting, motion correction, spatial smoothing (6 mm full width at half maximum Gaussian kernel), linear trend removal and temporal filtering (band pass 0.01-0.08 Hz) using FSL (FMRIB, Oxford University, UK). Nuisance sources of variance including white matter, CSF, and global mean signal were removed using regression [54]. Moreover, to further control motion artifacts, volumes with a threshold of signal change < 0.5% and a frame-wise displacement < 0.5 mm was discarded [55]. After rigid alignment of rsfMRI images to its structural image for each subject, spatial normalization of rsfMRI images to MNI template was achieved using the transformation field acquired during the structural image registration step [56–58]. Automated Anatomical Labeling atlas (AAL) [47] was used to parcellate the whole brain into 116 regions (45 bilateral cortical regions, 9 bilateral cerebellar regions and 8 vermis regions).

### Whole-brain functional connectivity analysis

Using MATLAB R212b (The Mathworks, Inc.), the mean time course of each AAL-defined region was obtained and Pearson’s correlation coefficient was calculated between all pairs of regions over the entire brain. A regional functional connectivity matrix was obtained (116 × 116 symmetric matrix) independently for SCA7 patients and healthy controls. Two-tailed two-group t-tests were performed for all pair-wise functional connections between SCA7 group and normal controls to detect whole-brain abnormal functional connections. Is well known that reducing the number of classification features accelerates computation and diminishes noise [24, 48, 59]. To this end, we remove the 116 diagonal values and only selected connections with a p value < 0.001 uncorrected in the lower triangular part of the symmetric matrix. In this step the multiple comparison correction is not required because this is just a feature selection method for the classification step [18]. Finally, we used each connection functional connectivity values and the behavioral data (CAG expansion, symptoms onset and SARA) to calculate the correlation between those variables.

### Support vector machine classification

In order to discriminate between SCA7 group and healthy controls we used the abnormal functional connections to feed the SVM classifier (linear kernel and sequential minimal optimization). In order to test the performance of this approach we used a cross-validation technique, in which, the data is split in k-folds to test k times the classifier using different instances to train and test. Classification accuracy, sensitivity (percentage of patients correctly classified) and specificity (percentage of controls correctly classified) were calculated based on a 10 fold cross-validation [48].

## Electronic supplementary material

Additional file 1: Figure S1: Significant correlations between functional connectivity and behavioral scores in the connections of left middle frontal gyrus and the right superior frontal gyrus. (PDF 97 KB)

## Abbreviations

SCA7: Spinocerebellar Ataxia type 7
ADCA: Autosomal Dominant Cerebellar Ataxia
CAG: Cytosine-adenine-guanine
rsfMRI: Resting state functional MRI
SVM: Support Vector Machines
EPI: Echo Planar Imaging
AAL: Automated Anatomical Labeling
MNI: Montreal Neurological Institute
SARA: Scale for the Assessment and Rating of Ataxia.
