# Supplementary material for: Whole-brain connectivity analysis and classification of spinocerebellar ataxia type 7 by functional MRI
Source: Cerebellum Ataxias. 2014 Jun 16;1:2. doi: 10.1186/2053-8871-1-2 (PMC4549137; doi:10.1186/2053-8871-1-2)
Supplement: Supplementary file 1 — Additional file 1: Figure S1: Significant correlations between functional connectivity and behavioral scores in the connections of left middle frontal gyrus and the right superior frontal gyrus. (PDF 97 KB) [file 40673_2013_2_MOESM1_ESM.pdf]

Supplementary Figure 1. Significant correlations between functional connectivity and behavioral scores in the connection of left middle frontal gyrus and the right superior frontal gyrus.

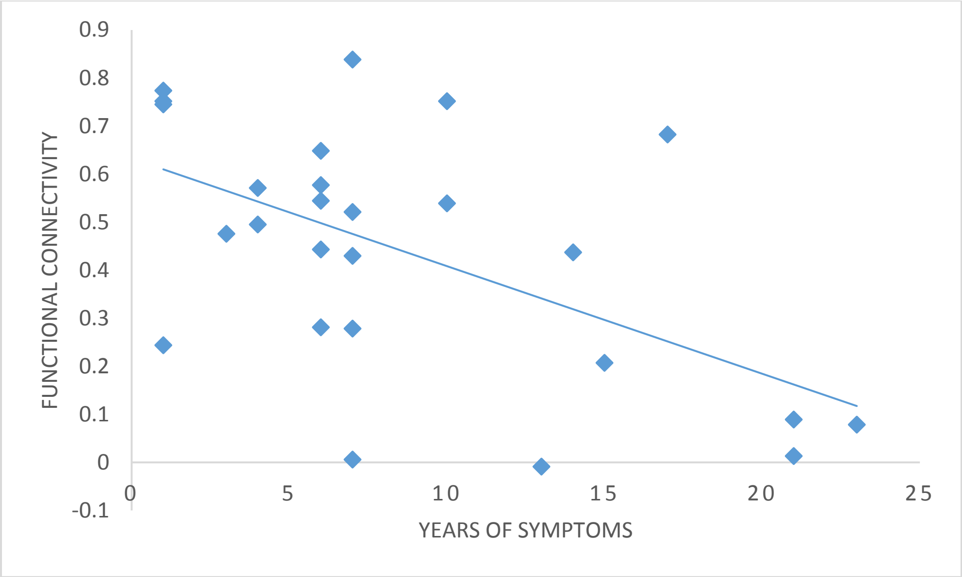

r = -0.55 p = 0.003

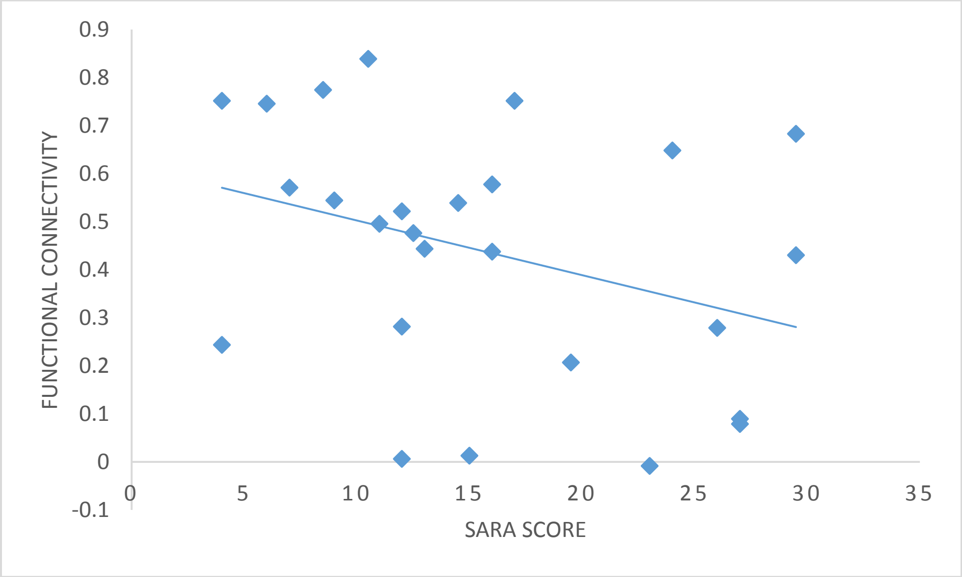

r = -0.34 p = 0.048
